# Elevated GRHL2 Imparts Plasticity in ER-Positive Breast Cancer Cells

**DOI:** 10.3390/cancers16162906

**Published:** 2024-08-21

**Authors:** Christy Zheng, Kaelyn O. Allen, Tianrui Liu, Natalia M. Solodin, Mark B. Meyer, Kelley Salem, Phillipos K. Tsourkas, Sean J. McIlwain, Jessica M. Vera, Erika R. Cromwell, Mary Szatkowski Ozers, Amy M. Fowler, Elaine T. Alarid

**Affiliations:** 1 McArdle Laboratory for Cancer Research, Department of Oncology, Carbone Comprehensive Cancer Center, University of Wisconsin-Madison, Madison, WI 53705, USA; 2 Department of Nutritional Sciences, University of Wisconsin-Madison, Madison, WI 53706, USA; 3 Department of Radiology, University of Wisconsin-Madison, Madison, WI 53792, USA; 4 Department of Biostatistics and Medical Informatics, University of Wisconsin-Madison, Madison, WI 53705, USA; 5 Proteovista LLC, Madison, WI 53719, USA; 6 Department of Medical Physics, University of Wisconsin-Madison, WI 53705, USA; 7 Carbone Comprehensive Cancer Center, University of Wisconsin-Madison, Madison, WI 53705, USA

**Keywords:** EMT, epithelial, mesenchymal, hybrid EMT, tumor progression, stemness, dormancy

## Abstract

**Simple Summary:**

High levels of the transcription factor Grainyhead-like protein 2 (GRHL2) contribute to worse outcomes for patients with breast cancer tumors that express estrogen receptor (ER). Using multiple methods to increase GRHL2 expression in breast cancer cells, we found that high GRHL2 promotes both epithelial and mesenchymal phenotypes. This is indicative of an intermediate state between epithelial and mesenchymal cells, which is associated with metastasis. We also observed that elevated GRHL2 stimulates properties characteristic of cellular plasticity, including a decrease in cellular proliferation with a reciprocal increase in dormancy and stem cell markers. Elevated levels of GRHL2 broadly changed its control of gene expression at the level of DNA and shifted nearby transcription factor binding sites from those associated with development to those associated with disease progression. These results provide a possible explanation for how elevated GRHL2 levels could contribute to poorer prognosis in ER-positive breast cancer.

**Abstract:**

Estrogen receptor (ER)-positive breast cancer is characterized by late recurrences following initial treatment. The epithelial cell fate transcription factor Grainyhead-like protein 2 (GRHL2) is overexpressed in ER-positive breast cancers and is linked to poorer prognosis as compared to ER-negative breast cancers. To understand how GRHL2 contributes to progression, GRHL2 was overexpressed in ER-positive cells. We demonstrated that elevated GRHL2 imparts plasticity with stem cell- and dormancy-associated traits. RNA sequencing and immunocytochemistry revealed that high GRHL2 not only strengthens the epithelial identity but supports a hybrid epithelial to mesenchymal transition (EMT). Proliferation and tumor studies exhibited a decrease in growth and an upregulation of dormancy markers, such as *NR2F1* and *CDKN1B*. Mammosphere assays and flow cytometry revealed enrichment of stem cell markers CD44 and ALDH1, and increased self-renewal capacity. Cistrome analyses revealed a change in transcription factor motifs near GRHL2 sites from developmental factors to those associated with disease progression. Together, these data support the idea that the plasticity and properties induced by elevated GRHL2 may provide a selective advantage to explain the association between GRHL2 and breast cancer progression.

## 1. Introduction

Breast cancer persists as the leading cancer diagnosed in women and the second leading cause of cancer-associated death [1,2]. This is largely attributed to estrogen receptor (ER)-positive breast cancer, which composes the majority of breast cancers [2]. Though ER-positive breast cancer is linked to higher 5-year survival rates relative to hormone receptor-negative breast cancers, the long-term mortality of patients with ER-positive tumors is higher than patients with ER-negative tumors 7 years post-diagnosis [2,3]. Patients with ER-positive breast cancer also experience a higher risk of late recurrence despite appropriate adjuvant therapies at initial tumor diagnosis [4,5,6]. These statistics underline the need for further research into understanding the mechanisms through which ER-positive breast cancer recurs over time.

In studies of phosphorylated ER (pER), an activated form of ER present in both primary and metastatic breast cancers, our group identified the nuclear transcription factor Grainyhead-like protein 2 (GRHL2) as a cooperating factor [7,8]. GRHL2 belongs in the Grainyhead family of transcription factors, originally discovered in *Drosophila melanogaster*, which is responsible for adhesion and developmental regulation [9,10]. GRHL2 is an epithelial cell marker vital in developmental processes through direct regulation of claudin-4 and E-cadherin [11,12,13,14]. Previous studies emphasize GRHL2’s canonical role in the maintenance of the epithelial identity and suppression of the epithelial to mesenchymal transition (EMT) through multiple avenues [11,15,16]. GRHL2 can repress the mesenchymal transcription factor ZEB1 and TGF-β signaling pathways to prevent EMT [15]. This points to the established notion that GRHL2 behaves as a tumor suppressor in various cancers and regulates tumor progression by inhibition of EMT [15,17,18,19]. Examination of primary breast cancers showed an association of GRHL2 protein expression with ER-positive but not ER-negative breast cancers [20,21,22]. GRHL2 is located on chromosome 8q22.3, a region frequently amplified in breast cancer [23]. Further studies showed that loss of GRHL2 led to decreased growth of tamoxifen-resistant ER-positive breast cancer cells and promoted migratory behavior [7,24].

Paradoxically, clinical evidence presents contradictions to GRHL2’s canonical role. Clinical data gathered from patients with ER-positive breast cancer not only reveal increased GRHL2 in breast tumors compared to normal tissues, but emphasize an association between high levels of GRHL2 expression and reduced disease-free survival [7,16,25,26]. Another study emphasized the correlation between GRHL2 and highly metastatic breast cancer cells, again reiterating unfavorable outcomes associated with increased GRHL2 expression [11]. Despite the association between high GRHL2 expression and poorer prognosis of ER-positive breast cancer, previous studies only focused on understanding GRHL2 in the context of the loss of function models. Given that GRHL2’s detrimental role in breast cancer progression remains unknown, in this study we examined the consequences of elevated levels of GRHL2 expression on ER-positive breast cancer cell biology and uncovered new roles which may contribute to its pro-oncogenic role in ER-positive breast cancer.

## 2. Materials and Methods

### 2.1. Cell Culture

MCF7 and MDA-MB-231 cells were maintained in Dulbecco’s Modified Eagle Medium (DMEM; Gibco, Waltham, MA, USA, #11965-092) supplemented with 10% Hyclone fetal bovine serum (FBS; Cytiva, Marlborough, MA, USA, #SH30910.03), 1% sodium pyruvate (Gibco, #11360-070), and 1% penicillin streptomycin (Pen/Strep; Gibco, #15140-122) in 10% CO_2_ at 37 °C. MCF7 RTA16 cells (P) and RTA16 tetracycline-inducible GRHL2-GFP (tet-inducible) (OE) were maintained as described above, but instead supplemented with 10% FBS sensitive to tetracycline-inducible models (VWR, Radnor, PA, USA, #97068-085) [27]. CAMA-1 and T47D cells were maintained in Roswell Park Memorial Institute 1640 medium (RPMI; Gibco, #11875-093) supplemented with 10% FBS, 1% Pen/Strep, 1% sodium pyruvate, and 25 nM Hepes (Gibco, #15630-080) in 5% CO_2_ at 37 °C. All cell lines were authenticated by Short Tandem Repeat Analysis when compared to ATCC commercially available cell lines. MDA-MB-231, CAMA-1, and T47D cells were obtained from ATCC. MCF-7 and MCF-7 RTA16 (P) were obtained from Dr. Craig Jordan and Dr. Adrian Lee, respectively.

### 2.2. Generation of Tetracycline-Inducible GRHL2-GFP Expression in MCF7 Cell Line

A tet-inducible cell line capable of expressing GRHL2-GFP upon exposure to doxycycline hyclate (Dox; Sigma-Aldrich, Burlington, MA, USA, #D9891) was engineered. A tet-inducible construct was created using a GRHL2-GFP plasmid (Origene, Rockville, MD, USA, #RG214498) subcloned into a pUHD10-3 backbone downstream of the tet response element [27,28]. GRHL2-GFP-inducible MCF7 cell clones were generated by stable co-transfection of GRHL2-GFP pUHD10-3 plasmid with pBabe-puro (Addgene, Watertown, MA, USA, #1764) with Lipofectamine™ 2000 (Thermo Fisher Scientific, Waltham, MA, USA, #11668-019) in the parental cell line MCF7 RTA-16. Positive clones were selected using 1 μg/mL puromycin (Sigma-Aldrich, P7255) and verified using Western blotting. To mitigate a potential clonal artifact, a Biosciences FACSAria™Cell Sorter was used to generate a pool of highly inducible GRHL2-GFP clones with minimal background, termed FACS GFP-positive tet-inducible GRHL2-overexpressing cells (OE pool). To visualize in vivo tumor xenografts, OE cells were infected with a constitutively active luciferase plasmid by lentiviral transduction.

### 2.3. Drug Treatment

Cells were cultured for 3 days in phenol-red-free DMEM or RPMI (Gibco, #31053-028 or #11835030, respectively) supplemented with 10% 6× charcoal stripped FBS, 1% sodium pyruvate, 1% Pen/Strep, and 2% L-glutamine (Gibco, #25030-081). Serum stripped DMEM or RPMI medias (ssDMEM or ssRPMI) were used to reduce non-polar, lipophilic compounds as estrogen deprivation media. In addition, these media synchronized cells in the cell cycle to mitigate growth differences. Cells were then treated with Dox and/or 10 nM 17β-estradiol (E2; Millipore Sigma, Burlington, MA, USA, #E2257) as per individual experimental conditions. Freshly prepared Dox solutions were only used for 7 days. Dox concentration and duration of treatment are specified for each experiment.

### 2.4. RNA Isolation and RT-qPCR

RNA isolation and reverse transcription quantitative real-time PCR (RT-qPCR) were performed as previously described [7]. Absolute quantification of *GRHL2* RNA levels were completed using standard curves previously performed by our group [7]. RT-qPCR primers used were as follows: for *GRHL2*: 5′-GCCACCAAATCTCTCCGTCA-3′ (forward) and 5′-CCACCATCACCACACTCCTG-3 (reverse); for *CDH1*: 5′-TATTGAAAGAGAAACAGGATGG-3′ (forward) and 5′-GGATGGTGTAAGCGATGG-3 (reverse); for *CLDN4*: 5′-TCTCCTCTGTTCCGGGTAGG-3′ (forward) and 5′-CGTCCATCCACTCTGCACTT-3 (reverse); for *UTRN*: 5′-CTGTGGATGATCGCCTTAAA-3′ (forward) and 5′-CTGGACTGACGTAGAGAGAA-3 (reverse); for *SCD2*: 5′-TCGGCGGAGTCGAGAGC-3′ (forward) and 5′-GCAGAAGCGTAGTCATCGTCA-3 (reverse); for *PEA15*: 5′-CTAGGGGAGGGGGCTGAGTT-3′ (forward) and 5′-GGTGGGGGTTGAGTGGTCTC-3 (reverse); for *NR2F1*: 5′-GCCTCAAAGCCATCGTGCTG-3′ (forward) and 5′-CCTCACGTACTCCTCCAGTG-3 (reverse); for *CDKN1B*: 5′-GGCTAACTCTGAGGACAC-3′ (forward) and 5′-TTCTTCTGTTCTGTTGGC-3 (reverse); for *GFP*: 5′-CGATCTGGATGGCAGCTTCA-3′ (forward) and 5′-TTGAAGGCGTGCTGGTACTC-3 (reverse); for *VIM*: 5′-ACACCCTGCAATCTTTCAGACA-3′ (forward) and 5′-GATTCCACTTTGCGTTCAAGGT-3 (reverse).

### 2.5. RNA Sequencing

OE cells were synchronized, estrogen-deprived, and treated with 1 μg/mL Dox for 72 h, with media changes every 24 h. On day 3 of treatment, cells treated with Dox were sorted for GFP expression using a Biosciences FACSAria™Cell Sorter. Cells treated with vehicle were not sorted. RNA sequencing was performed by AZENTA Life Sciences Next Generation Sequencing. Reads were aligned to the *Homo sapiens* GRCh38 reference genome with STAR aligner, and counts were generated with FeatureCounts. Batch effects were addressed using the ComBat_seq function in the R package “sva”. Differential expression testing was carried out with DESeq2, with log foldchange shrinkage (type = “ashr”). Differentially expressed genes were identified with FDR < 0.05. Gene set analysis was carried out on the GSVA R package and the GO BP sets downloaded from MsigDb. Differential analysis of GSVA results was performed with limma and FDR < 0.05. RNA isolated by AZENTA and parallel samples were used for RT-qPCR validation.

### 2.6. Protein Preparation and Western Blotting

Protein preparation and Western blotting were performed as previously described using antibodies for GRHL2 (Millipore Sigma, #HPA004820), estrogen receptor alpha (ERα clone sc-7207; Santa Cruz Biotechnology, Santa Cruz, CA, USA, #H-184), and β-actin (clone AC-15; Millipore Sigma, #A5441) [7]. Blots were visualized with Amersham™ ECL Western blotting detection reagents (Cytiva, #RPN2106) and Clarity Western ECL substrate (Bio-Rad, Hercules, CA, USA, #170-5061) on an Azure Imaging System 600 (Azure Biosystems, Dublin, CA, USA).

### 2.7. Chromatin Immunoprecipitation (ChIP) and Sequencing

OE cells were synchronized, then 5.0 × 10^6^ cells were seeded in 10 cm plates with ssDMEM supplemented with estradiol. Cells were simultaneously treated with either 1 μg/mL Dox or vehicle for 24, 48, or 72 h time points, with media changes every 24 h.

Samples were harvested and prepared for ChIP as previously described, with the exception that sonication conditions consisted of 20 s of sonication at 7% maximum intensity for a total of three rounds [8]. Precleared samples were immunoprecipitated using GRHL2 (Millipore Sigma, #HPA004820) or H3K27 acetylation (Abcam, Cambridge, UK #ab4729) antibodies. ChIP qPCR was performed using 1 μL purified DNA and primers for *NR2F1*: 5′-AATTAACCAGTGCCTGGGCG-3′ (forward); 5′-CGGGCTCTGAATCTGTCTGG-3′ (reverse), *VIM* promoter: 5′-GTCCAGTCCTCTGCCACTCT-3′ (forward); 5′-GTCCAGTCCTCTGCCACTCT-3′ (reverse), *SP6*: 5′-TCTCTGGATCACCGGCTAGT-3′ (forward); 5′-TTAGAACCCCGATGGAGGGA-3′ (reverse).

Prior to creating libraries in preparation for sequencing (ChIP-seq), DNA concentrations were measured with the Qubit dsDNA Quantitation High Sensitivity assay kit (Thermo Fisher, #Q32851). Bioinformatics on ChIP-seq data and peaks were called and processed as previously reported [29]. Briefly, ChIP libraries were generated using the NEBNext Ultra II DNA Library Prep Kit for Illumina (NEB, Ipswich, MA, USA, #E7645) and NEBNext Multiplex Oligos for Illumina (NEB, #6440). Samples were sequenced on the NovaSeqX Plus (Azenta/Genewiz) with 150 bp paired end sequencing with a target of 30 million reads per sample (minimum). We performed bioinformatic analysis of the sequencing reads using Bowtie2 to map the reads to the hg38 genome and HOMER to process peaks, perform de novo motif discovery and analysis, and for visualization of the tracks in the UCSC genome browser [30,31].

### 2.8. Transient Transfection

Transient transfections were used as an orthogonal approach to model GRHL2-GFP overexpression in additional ER-positive cell lines. CAMA-1, T47D, and MCF7 cells were plated in 6-well plates at a density of 5.0–8.0 × 10^5^ cells in Pen/Strep-free RPMI or DMEM (MCF7). Cells were transfected using Lipofectamine™ 3000 or FuGENE™ HD Transfection Reagent (FuGENE; Promega, Fitchburg, WI, USA, #E2311) as per manufacturer’s instructions with plasmids containing pCMV6-AC-GFP (control) (Origene, #PS100010) or GRHL2-GFP. GFP was expressed in the vector control to ensure overexpressing GRHL2 tagged with GFP-induced phenotypes were not artifacts of elevated GFP levels. A CMV-tk-Luc+ plasmid was co-transfected for transfection efficiency normalization in downstream analyses. Cells were incubated under the conditions described above for 48 h prior to harvesting, with a media change after 24 h.

### 2.9. Migration Assay

MCF7 and CAMA-1 cells were plated at a density of 7.5 × 10^5^ cells/well in a 6-well plate and transfected with GRHL2-GFP plasmid according to the transfection procedure described above. Estrogen-deprived OE and P cells were cultured in ssDMEM and induced with 0.5 μg/mL Dox or vehicle prior to seeding in migration assays. After 24 h of transfection and/or treatment, cells were seeded in chambers as previously described using Ibidi chambers [7]. Migration into the cell-free gap was imaged at 0 h and again after 24 h. The gap width was measured using the Wound Healing Size Tool plugin for ImageJ [32].

### 2.10. Flow Cytometry

Cells were dissociated with 0.05% trypsin-EDTA (Gibco, #15400-054), neutralized with trypsin inhibitor (Sigma-Aldrich, #T6414), and resuspended in phenol-red-free DMEM supplemented with 1% bovine serum albumin (BSA; Sigma-Aldrich, #A1470). Single cell suspension was achieved by resuspending with a 23-gauge needle and 40 μm filter. For cell cycle analyses, 2.5 × 10^5^ cells were fixed with cold 100% ethanol and stained with propidium iodide (PI; Sigma-Aldrich, #P4170) staining solution (100 μg/mL RNase A [Invitrogen, Carlsbad, CA, USA, #12091021] and 50 μg/mL PI in 1× phosphate-buffered saline [PBS]). A Thermo Fisher Attune NxT flow cytometer was used to capture PI dye intensity. Events were analyzed with ModFit software (version 5). For stem cell marker analysis, cells were treated as described and samples were incubated with AlexaFluor conjugated fluorescent antibodies: 20 μL CD24-PE (Fisher, #BDB555428, ML5) or 1 μL CD44-APC (Fisher, Waltham, MA, USA, #17-0441-82, IM7) on ice for 30 min in the dark. Cells were washed with 1× PBS and resuspended in 100 μL serum-, supplement- and phenol-red-free DMEM. Then, 1 μg/mL DAPI was included to distinguish live/dead cells. Biosciences FACSAria™Cell Sorter was used to differentiate CD24 and CD44 populations while simultaneously visualizing GRHL2-GFP-overexpressing cells. For ALDH1 detection, cells were subjected to the AldeRed ALDH Detection Assay (Millipore, Burlington, MA, USA, #SCR150) with diethylaminobenzaldehyde (DEAB)-treated cells as a control to inhibit ALDH activity. To determine appropriate gating for CD24 and CD44 quadrants, stained parental MCF7 and MDA-MB-231 cells were used to differentiate between CD24+/CD44− and CD24−/CD44+, respectively, and to establish gating parameters for all samples. In addition, MDA-MB-231 cells were used for gating ALDH+ parameters.

### 2.11. Immunocytochemistry and Immunohistrochemistry

The expression of various proteins was indirectly evaluated with immunostaining. Immunocytochemistry was performed in multi-well chamber slides (Thermo Fisher Scientific, #12-565-7) coated with 0.02% gelatin (Sigma-Aldrich, #G1393). Cells were treated as described, and fixed with warm 4% formaldehyde (Millipore Sigma, #F8775) for 15 min at room temperature, and pre-blocked with 5% goat serum (Sigma-Aldrich, #G9023) in 0.3% Triton. Slides were incubated with primary antibodies E-cadherin (E-cad cl. 36; BD Biosciences, #BDB610181), vimentin (VIM cl. E-5; Cell Signaling, Danvers, MA, USA, #5741), or GRHL2 (Millipore Sigma, #HPA004820). Slides were then incubated in AlexaFluor conjugated secondary antibodies: α-mouse 488 (Invitrogen, #A32723), α-mouse 568 (Invitrogen, #A-11004), and α-rabbit 680 (Invitrogen, #A32734) diluted 1:200 in antibody dilution buffer in the dark for 1 h at room temperature. Slides were mounted with Prolong Gold Antifade Reagent with DAPI (Invitrogen, #P36931). Immunohistochemistry on tumor samples was performed with excised tumors fixed in 10% formalin, embedded in paraffin, and sectioned. After deparaffinization and rehydration, antigen retrieval was achieved using 10 mM sodium citrate (pH = 6) for 30 min at 95 °C in a Decloaking Chamber™ NxGen (Biocare Medical, #DC2012). Slides were blocked for 45 min in 5% goat serum (Vector Laboratories, Newark, CA, USA, #PK-4001) in TBST (50 mM Tris-HCl [pH 7.4], 150 mM NaCl, 0.1% Tween 20) prior to an overnight incubation at 4 °C with a primary antibody for GRHL2 (Millipore Sigma, #HPA004820), estrogen receptor alpha (ERα cl. SP1; Invitrogen, #MA5-14501), p27 (Invitrogen, #PA5-27188), vimentin (VIM cl. E-5; Cell Signaling, #5741), or Ki-67 (Invitrogen, cl. SP6, #MA5-14520) diluted 1:100 in blocking solution. Slides were incubated with a secondary antibody (α-mouse or α-rabbit; Vector Laboratories, #PK-4002, #PK-4001) at room temperature for 60 min, then in 1× ABC reagent using the Vectastain ABC Kit for 45 min. Tumor sections were stained with DAB reagents 1 and 2 (Vector Laboratories, #30107 and #30091) in a 1:1 ratio until a definitive color change was observed, then counterstained with hematoxylin (Vector Laboratories, #H-3404) for 5 s before dehydration. Whole slides were scanned at 20× magnification using an Aperio Digital Pathology Slide Scanner and analyzed at 10× magnification with the Aperio ImageScope.

### 2.12. Soft Agar Assay

Cells were assessed for 3D colony formation and proliferation through the use of a soft agar assay. OE and P cells were cultured in ssDMEM with 10 nM E2. Cells were seeded at a density of 1.0 × 10^4^ cells/well in 0.4% SeaPlaque™ Agarose Lonza ssDMEM (Agarose; Fisher, BMA50101) with 1 μg/mL Dox in 6-well plates with a prepared 0.8% agarose ssDMEM bottom layer. The soft agar layers were created as previously [33]. For 14 days, cells were incubated in 5% CO_2_ at 37 °C until colonies were visualized via microscopy, with additional 1 μg/mL Dox treatments every 4 days. Colonies were counted by staining wells with 0.005% crystal violet solution in methanol (Millipore Sigma, #C0775). Images were captured via fluorescence microscopy and bright field microscopy. Analysis of soft agar colonies was conducted with ImageJ.

### 2.13. Mammosphere Assay

Primary and secondary mammosphere assays were performed to determine the self-renewal capacity of cells. Estrogen-deprived OE and P cells were seeded in ultra-low attachment wells with formulated mammosphere medium. For primary mammospheres, 400 cells in single cell suspension were seeded in 96-well ultra-low attachment plates (Corning, #3474) with formulated mammosphere medium supplemented with 1 μg/mL Dox or vehicle treatment. All cells were simultaneously supplemented with E2 to support growth at time of seeding. To prevent aggregate formation, no additional treatments were included, and physical handling was minimized. After 14 days of incubation in 5% CO_2_ at 37 °C, mammospheres were imaged via fluorescence microscopy to determine mammosphere formation efficiency. For secondary mammospheres, primary mammospheres were enzymatically dissociated, pelleted, and resuspended in fresh, formulated mammosphere medium to ensure single cell suspension. Then 2.0 × 10^4^ cells were seeded in 24-well Nunclon™ Sphera™super-low attachment plates (Thermo Fisher Scientific, #174930) with E2 supplemented mammosphere medium and 1 μg/mL Dox or vehicle.

### 2.14. Tumor Xenograft

Ten athymic nude female mice were subcutaneously implanted with a 20 μg (60-day release) silastic estradiol capsule [34]. After one week, mice were injected bilaterally into the mammary fat pads with luciferase-expressing OE and P cells using a 1:1 mixture of 5.0 × 10^6^ cells and Matrigel (Corning, Corning, NY, USA, #354234). Pilot studies showed differences in tumor growth prior to treatment. Hence, P and OE cells were injected at 2-week intervals from each other to achieve similar tumor volume sizes at time of Dox treatment. Silastic capsules were replaced on day 60. Once palpable tumors formed, the tumor size was measured every two days with calipers and tumor volume was calculated by the formula of width^2^ × length/2. At time of sacrifice, tumors were flash frozen or formalin fixed. All studies were carried out under approved protocols by the Office of Biological Safety (B00000496) and the Institutional Animal Care and Use Committee (IACUC) at the University of Wisconsin–Madison (M005554).

### 2.15. Statistical Analysis

Data are mean ± standard error of the mean of biological triplicates. Statistical analysis was performed using GraphPad Prism (version 10.1.). Paired t-tests were used with a *p*-value < 0.05 considered significant.

## 3. Results

### 3.1. A Tetracycline-Inducible Model of High GRHL2 Expression in Breast Cancer Cells Expresses GRHL2 Protein and mRNA in a Dox Dose-Dependent Manner

**Figure 1:**
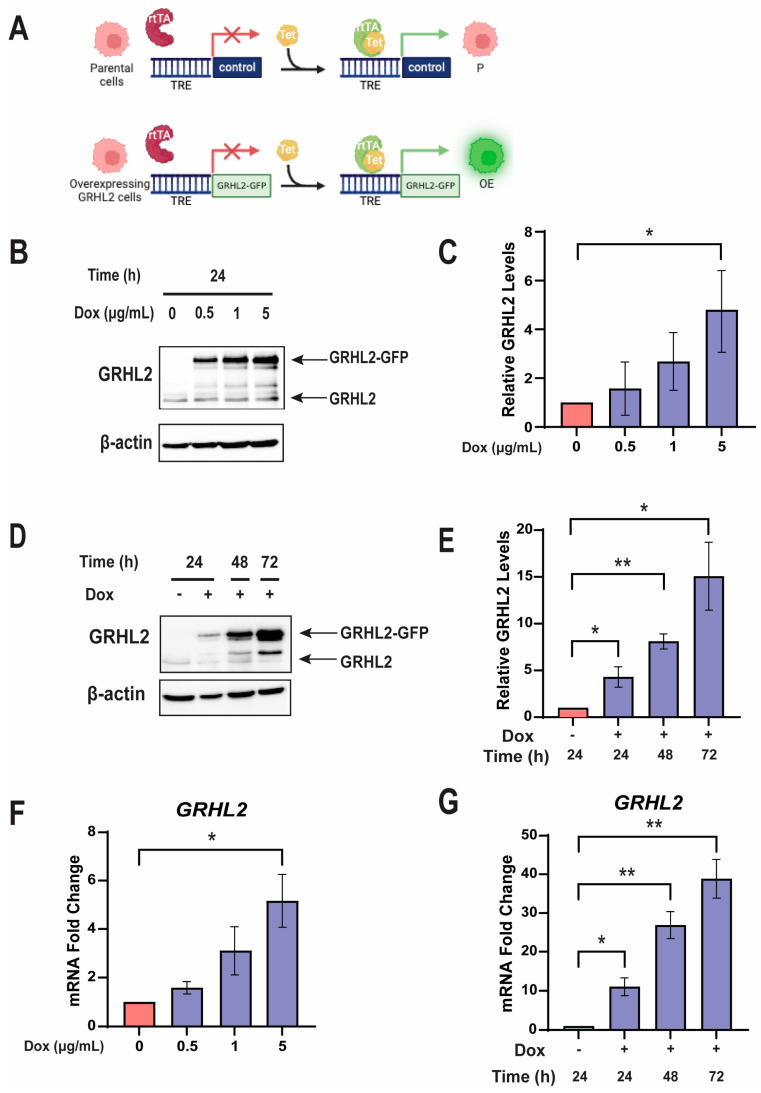
A tetracycline-inducible model of high GRHL2 expression in breast cancer cells expresses GRHL2 protein and mRNA in a Dox dose-dependent manner. (**A**) Schematic diagram of tet-inducible models. A Dox-inducible GRHL2-GFP construct was created via rtTA with a control or *GRHL2-GFP* plasmid in a pUHD10-3 backbone along with a tetracycline response element (TRE). Parental (P) cells lack an exogenous *GRHL2-GFP* gene. Cells with inducible overexpression of GRHL2-GFP are referred to as (OE). (**B**) Representative Western blot of engineered OE cells treated with Dox at the indicated doses. Endogenous GRHL2 (GRHL2) and overexpressed GRHL2 (GRHL2-GFP) are shown. β-actin is shown as a loading control. Uncropped Western blots are included in the Supplementary Materials. (**C**) Quantification of total GRHL2 protein (endogenous GRHL2 and GRHL2-GFP) in OE cells. Increase in total GRHL2 levels was relative to the amount of endogenous GRHL2 protein present in cells grown in the absence of Dox, set at 1.0. n = 3. *, *p* < 0.05. (**D**) Representative Western blot as in (**B**) in OE cells treated for the indicated length of time with Dox. Uncropped Western blots are included in the Supplementary Materials. (**E**) Quantification of GRHL2 protein in OE cells treated with 1 μg/mL Dox for the indicated length of time. Increase in total GRHL2 levels was relative to the amount of endogenous GRHL2 protein present in cells grown in the absence of Dox, set at 1.0. n = 3. *, *p* < 0.05; **, *p* < 0.01 relative to vehicle. (**F**) RT-qPCR analysis of *GRHL2* in dose response studies. n = 3. *, *p* < 0.05 relative to vehicle. (**G**) RT-qPCR analysis of *GRHL2* in time course studies. n = 3. *, *p* < 0.05; **, *p* < 0.01 relative to vehicle.

In order to investigate the effects of high levels of GRHL2 in ER-positive breast cancer cells, a tetracycline-inducible GRHL2-GFP (OE) overexpression model was created in MCF7 cells in which a GRHL2-GFP fusion protein is produced upon induction with doxycycline (Dox) (Figure 1A) [27]. Prior to any functional assays, the model was characterized for optimal protein and mRNA expression. Dose response assays show a dose-dependent increase in GRHL2 protein (Figure 1B,C). Though maximal induction occurred at 5 μg/mL of Dox, this dose is known to compromise viability [35,36]. Therefore, tet-inducible cells were treated with 1 μg/mL of Dox at 24 h intervals for 72 h. GRHL2-GFP protein progressively increases after repeatedly administering Dox during a 72 h period in a time-dependent manner. The increase in protein levels over time is significant and maximized after 72 h (Figure 1D,E).

Similar trends in mRNA were observed in both the dose response and time course studies. Significantly higher levels of *GRHL2* mRNA transcripts were found with 5 μg/mL of Dox treatment, although *GRHL2* mRNA showed a trending increase with Dox concentration (Figure 1F). After treatment with 1 μg/mL of Dox, *GRHL2* mRNA levels were significantly higher over time up to 72 h (Figure 1G). In light of the significant increases in GRHL2 protein and mRNA in time course studies and to mitigate the potential toxicity of Dox, a dose of 1 μg/mL Dox was used in subsequent experiments.

### 3.2. High GRHL2 Expression Increases Epithelial Cell Phenotypes

**Figure 2:**
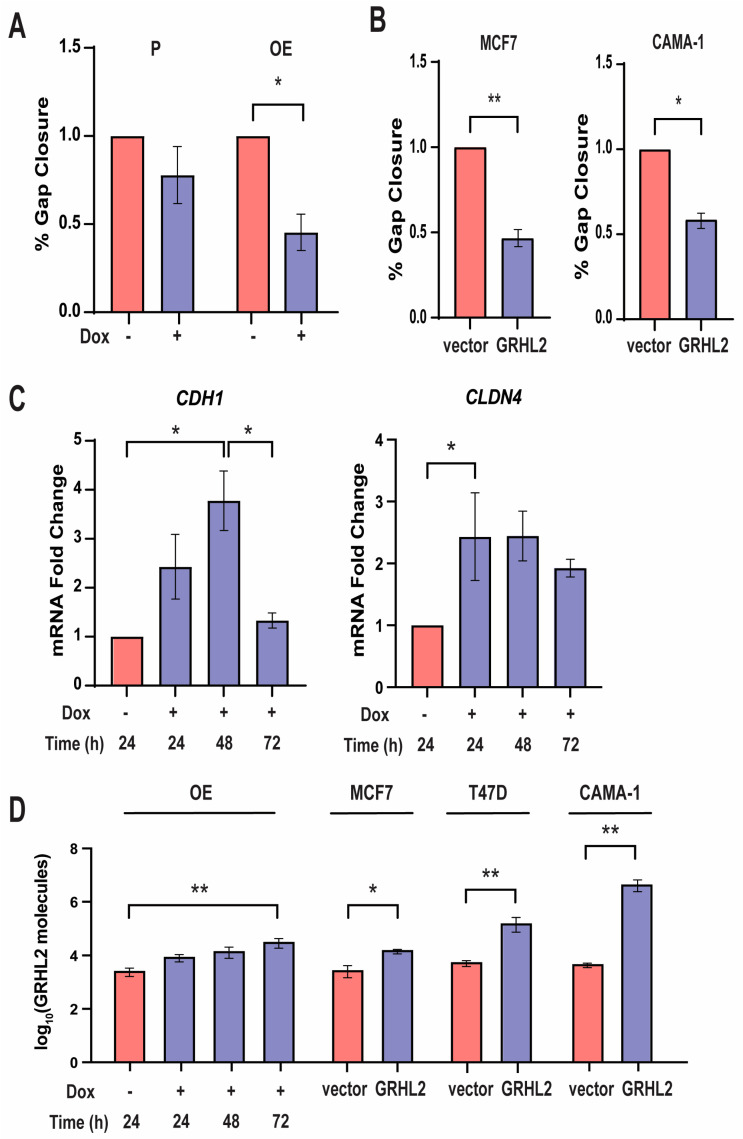
High GRHL2 expression increases epithelial cell phenotypes. (**A**) Quantification of the % cell gap closure of P and OE cells subjected to a migration assay. n = 3. *, *p*-value < 0.05 relative to vehicle. (**B**) Quantification of the % cell gap closure over 24 h of MCF7 and CAMA-1 cells transiently transfected with *GRHL2* DNA. n = 3. *, *p*-value < 0.05 relative to vehicle; **, *p*-value < 0.01 relative to vector control. (**C**) RT-qPCR analyses of representative epithelial genes *CDH1* and *CLDN4* in OE cells. n = 3. *, *p*-value < 0.05 relative to no Dox. Supplementary Figure S1E supports increase in *CDH1* in T47D cells transfected with 1 μg/uL of GRHL2-GFP plasmid. (**D**) Absolute quantification of total *GRHL2* mRNA in OE cells and MCF7, T47D, and CAMA-1 cells transfected with *GRHL2* DNA. n = 3. *, *p* < 0.05; ** *p* < 0.01 relative to no Dox treatment or vector control. Supplementary Figure S1 provides fluorescence microscopy confirmation of elevated GRHL2 levels in these cells.

The Grainyhead family is well known for maintaining epithelial integrity in *Drosophila* and vertebrates [37,38]. Accordingly, previous work from our lab showed that a loss of GRHL2 increased migration in MCF7 cells [7]. Thus, we first asked whether high levels of GRHL2 would also impact cellular migration. A migration assay was conducted using parental (P) cells and OE cell lines treated for 24 h with 1 μg/mL of Dox prior to establishing a cell-free gap. Migration across this cell-free gap was significantly inhibited in cells with high levels of GRHL2 compared to cells without Dox and P cells over the course of 24 h (Figure 2A). To further validate this finding, MCF7 and CAMA-1 cells were transiently transfected with 1 μg GRHL2-GFP (GRHL2) for 24 h prior to a migration assay. In both cell lines, GRHL2-overexpressing cells migrated significantly less than those transfected with a vector control over the course of 24 h (Figure 2B). This indicates that GRHL2 overexpression acts as a blockade against migratory behaviors. Fluorescence microscopy was conducted on OE cells treated with Dox and transfected cells to verify the overexpression of GRHL2-GFP (see Supplementary Figure S1A–D).

The expression of both CDH1 (E-cadherin) and CLDN4 (claudin-4) is correlated with an epithelial phenotype through the establishment of tight cell–cell junctions [39,40]. Notably, CLDN4 is directly regulated by GRHL2, as GRHL2 binds and remodels the promoter [14]. OE cells treated with 1 μg/mL of Dox showed a significant increase in *CDH1* mRNA relative to an untreated control after 48 h. However, mRNA expression returned to nearly baseline level after 72 h of treatment. Similar treatment conditions yielded a significant elevation in *CLDN4* mRNA relative to an untreated control after 24 h (Figure 2C). Similar expression patterns of the same epithelial genes were also confirmed in T47D cells transfected with 1 μg GRHL2-GFP plasmid (see Supplementary Figure S1E). Absolute quantification of *GRHL2* mRNA confirmed that OE cells treated with Dox and cells transfected with a GRHL2-GFP plasmid expressed significantly higher levels of *GRHL2* mRNA (Figure 2D). Hence, mRNA analyses support GRHL2 behaving as an epithelial cell marker by upregulating known epithelial-related genes, and further indicate that increasing levels of GRHL2 does result in an enhanced epithelial phenotype and decreased migratory behavior.

### 3.3. GRHL2 Overexpression Alters Its Endogenous Transcriptional Activity and Gene Expression

**Figure 3:**
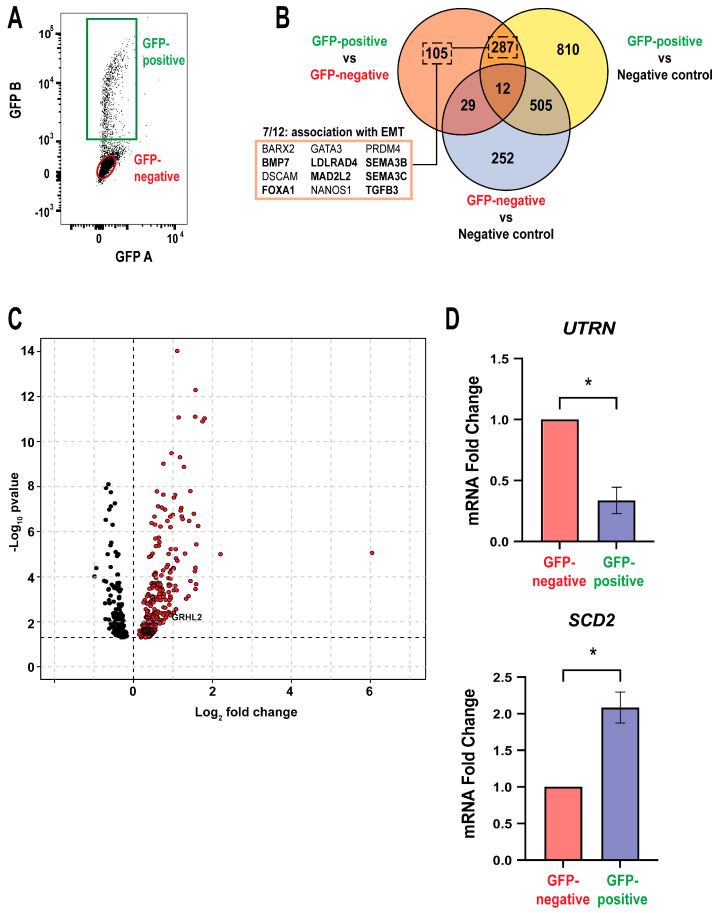
GRHL2 overexpression alters its endogenous transcriptional activity and gene expression. (**A**) Flow cytometry FACS gating to isolate GFPpositive, GRHL2-high cells (green) from GFP-negative, GRHL2-low (red) cells. FACS-sorted GFP-negative and -positive samples were used for RNA sequencing along with a negative untreated control. n = 5. (**B**) Venn diagram of RNA-seq data displaying the differentially expressed (DE) genes between the GFP-negative, GFP-positive, and negative control gene sets. Select genes are referenced, and bolded genes refer to an association with the epithelial to mesenchymal transition (EMT) gene ontology pathway. Supplemental Table S1 specifies the 105 DE genes in the Venn diagram. (**C**) Volcano plot of RNA-seq data depicts fold change of downregulated (black) and upregulated (red) DE genes. Fold change represents the GFP-negative vs. GFP-positive gene set comparison. Outlier genes include *ENDOD1* and *FAM41C*. (**D**) RT-qPCR validation of representative genes from the GFP-negative vs. GFP-positive gene set. n = 3. *, *p* < 0.05 relative to GFP-negative.

We next investigated if there were other genes and biological processes associated with GRHL2 overexpression. RNA-seq was performed on OE cells treated with 1 μg/mL Dox for 72 h. Given the heterogeneity of OE cells being induced and the dynamic nature of GRHL2 overexpression, FACS was utilized to isolate the cells actively overexpressing GRHL2-GFP in a Dox-treated population by sorting based on GFP signal intensity. Subsets of cells with elevated levels of GFP are denoted as GFP-positive (Figure 3A). GFP-negative cells were treated with Dox but did not show induction of the GRHL2-GFP fusion protein. RNA-seq was performed on GFP-positive, GFP-negative, and control cells not treated with Dox.

Differential expression analysis was performed using DESeq, and differentially expressed (DE) genes were identified with FDR < 0.05. As expected, when comparing Dox-treated samples to the untreated negative control, many DE genes arose from metabolic changes induced by doxycycline treatment (Figure 3B,C) [35]. Outlier genes associated with Dox treatment and others shared with negative control comparisons were deemed not regulated by GRHL2 overexpression, allowing us to confidently focus on the 433 genes impacted by GRHL2 overexpression (Figure 3C). Unsurprisingly, DESeq yielded DE genes previously known to be co-regulated with ER, such as *GATA3* and *FOXA1*, since these genes are associated with the epithelial phenotype in ER-positive breast cancer cells [41]. Upon further examination of the unique 105 genes captured from a true GRHL2 overexpression comparison, numerous genes associated with epithelial to mesenchymal transition (EMT) emerged and are highlighted (Figure 3B). EMT is the process by which breast cancer progresses from epithelial cells in the mammary glands to mesenchymal cells capable of migrating and invading distal tissues to ultimately establish metastases. This dynamic process is predominantly governed by transcription factors, which can modulate gene expression in favor of either an epithelial or mesenchymal identity [42,43,44]. It is speculated that the shifting interplay of transcription factors and resultant phenotypes may explain how breast cancer metastases continue to evade systemic treatments to propagate their own survival [45]. Notably, GRHL2 has previously been implicated as both inhibitor and activator of EMT in various cancer cell lines, and this activity appears to be context-dependent [46,47].

Validation studies by RT-qPCR on FACS-sorted cells showed downregulation of tumor suppressor *UTRN*, a gene that is recently linked to drug resistance and poor prognosis in breast cancer. Interestingly, the gene *SCD2*, which is primarily involved in fatty acid synthesis, is also increased by high levels of GRHL2. *SCD2* is associated with an increase in self-renewal, stemness, and tumorigenesis [48,49]. These data demonstrate that elevated expression of GRHL2 in ER-positive breast cancer cells alters gene expression beyond the established epithelial marker functions.

### 3.4. GRHL2 Overexpression Regulates Growth and Developmental Pathways

**Figure 4:**
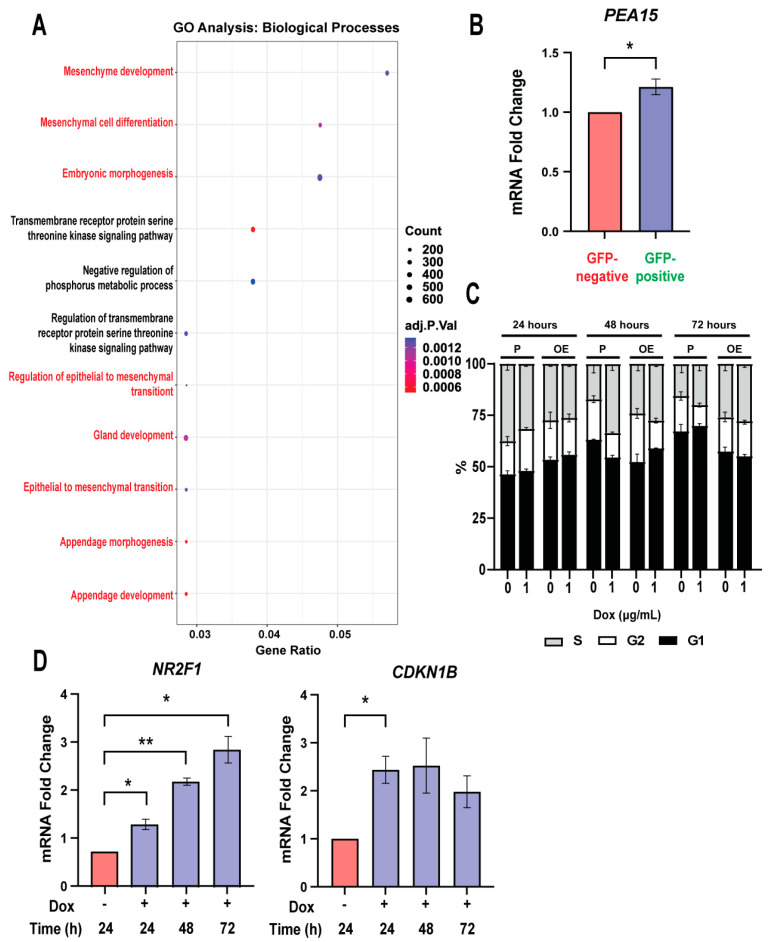
GRHL2 overexpression regulates development and growth. (**A**) Gene ontology analysis with MSigDB biological processes performed with clusterProfiler on the unique 105 gene cluster in the GFP-negative versus GFP-positive gene set. Terms related to EMT and development are highlighted in red. (**B**) RT-qPCR analysis of *PEA15* from the unique 105 gene cluster in the GFP-negative versus GFP-positive gene set. n = 3. *, *p* < 0.05 versus GFP-negative. (**C**) Flow cytometry cell cycle analysis shows % of cells in S, G2, and G1 cell cycles in P and OE cells. n = 3. (**D**) RT-qPCR analyses of representative tumor dormancy genes *NR2F1* and *CDKN1B* in OE cells. n = 3. *, *p*-value < 0.05; **, *p* < 0.01 versus no Dox.

To determine the biological processes regulated by GRHL2 overexpression, we performed gene ontology (GO) analysis using the Gene Set Variation Analysis and MSigDB, focusing on the 105 unique genes in the GFP-negative and GFP-positive subset (see Supplemental Table S1). As expected, genes related to GRHL2 overexpression were associated with epithelial-related terms and development. However, four of the top 11 most prominent gene ontology analysis pathways were related to mesenchyme development and EMT (Figure 4A). An example is the expression of *PEA15*. In triple negative breast cancer, PEA15 functions in supporting metastasis and EMT [50]. As expected, GRHL2 overexpression in GFP-positive OE cells upregulates *PEA15* expression when compared to the GFP-negative (Figure 4B). This supports the growing evidence that GRHL2 is related to EMT, though its role in regulating the mesenchymal identity in EMT is still not known. Another function of PEA15 is its regulation of proliferation, by which phosphorylated PEA15 blocks apoptosis and enhances anoikis-resistant growth in mammary cells [51,52,53,54]. Given GRHL2’s maintenance of the epithelial state and upregulation of proliferation-associated genes, we next asked if elevated GRHL2 levels changed proliferation by conducting cell cycle analysis in OE cells treated with 1 μg/mL of Dox over 24, 48, and 72 h. Interestingly, we saw no change in the composition of the population in terms of the percent of cells in the S, G2, and G1 cell cycles (Figure 4C, Supplemental Table S2). This lack of growth response indicates that, first, high GRHL2 expression does not alter cell cycle in 2D. Second, the data allude to the possibility that GRHL2 overexpression may be supporting a dormant, quiescent state, which is linked to mesenchymal-like programs [55].

To explore if dormancy may be stimulated by elevated GRHL2 expression, OE cells were treated with 1 μg/mL of Dox over 24, 48, and 72 h prior to RNA isolation and RT-qPCR analysis of dormancy-associated markers *NR2F1* and *CDKN1B* [56,57,58]. We saw an increase in mRNA expression of these dormancy markers in a dynamic manner, with the maximum expression of *NR2F1* seen in OE cells treated with Dox for 72 h (Figure 4D). Together, these findings imply that high expression of GRHL2 has a pleiotropic role in enriching both an epithelial and EMT gene program, while slowing growth and promoting expression of dormancy-associated genes.

### 3.5. GRHL2 Overexpression Inhibits Proliferation in Vivo

**Figure 5:**
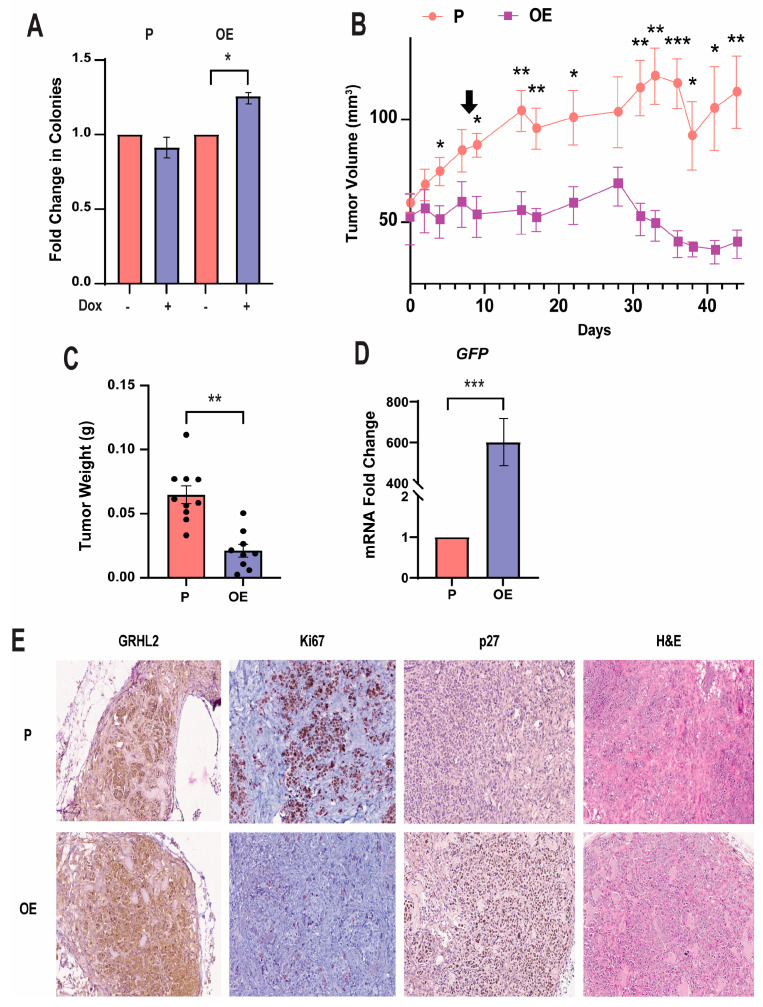
GRHL2 overexpression inhibits proliferation in vivo. (**A**) Quantification of soft agar colony formation in P and OE cells. n = 3. *, *p* < 0.05 versus no Dox. (**B**) Quantification of tumor growth in mice injected with P or OE cells. An arrow marks the introduction of Dox treatment. n = 10. *, *p* < 0.05; **, *p* < 0.01; ***, *p* < 0.001 versus parental. (**C**) Weight of tumors derived from P or OE tumors. n = 10. **, *p* < 0.01 versus P. (**D**) RT-qPCR analysis of *GFP* gene expression in murine tumors. n = 8. ***, *p* < 0.001 versus P tumors. (**E**) Representative immunohistochemistry (IHC) staining on proliferation and dormancy-associated proteins on excised P and OE tumors. IHC staining portrays: GRHL2, Ki67, p27, and hematoxylin and eosin (H&E) in tissue sections derived from mammary fat pad tumors. All images are shown at 10× magnification.

To further evaluate proliferation and the tumorigenic potential of GRHL2 overexpression, OE cells were treated with 1 μg/mL of Dox to observe anchorage-independent growth. No change in colonies was observed in P cells after Dox treatment, but a significant increase in colonies was observed in OE cells treated with Dox when compared to untreated P cells (Figure 5A). Hence, GRHL2’s effects differ in 3D vs. 2D environments. Interestingly, this anchorage-dependent growth response contradicts the previous cell cycle analysis and upregulation of dormancy markers. However, this could be explained by the dynamic nature of quiescence and how cells can exit dormancy depending on the tumor microenvironment [59].

To evaluate in vivo tumor growth, P and OE cells were injected unilaterally into the mammary fat pads of athymic, nude, female mice supplemented with estrogen and treated with Dox. Tumor volume measurements revealed that tumor growth was consistently and significantly stunted in tumors derived from OE cells, while the growth of P tumors appeared uninhibited (Figure 5B). Notably, OE tumors appeared to begin decreasing in volume at day 30 of this study. In agreement with the decrease in tumor volume, OE tumors were also found to weigh less than P tumors (Figure 5C). Further, OE tumors show decreased immunostaining for proliferative marker Ki67 by qualitative visual assessment. Consistent with the RNA-seq results, there was an increase in dormancy marker p27 relative to P tumors, while retaining characteristically higher levels of GRHL2 protein (Figure 5E). GRHL2 overexpression was confirmed in OE tumors treated with Dox by measuring *GFP* mRNA expression levels in OE and P tumors via RT-qPCR (Figure 5D).

### 3.6. GRHL2 Overexpression Enriches Stem Cell-like Characteristics

**Figure 6:**
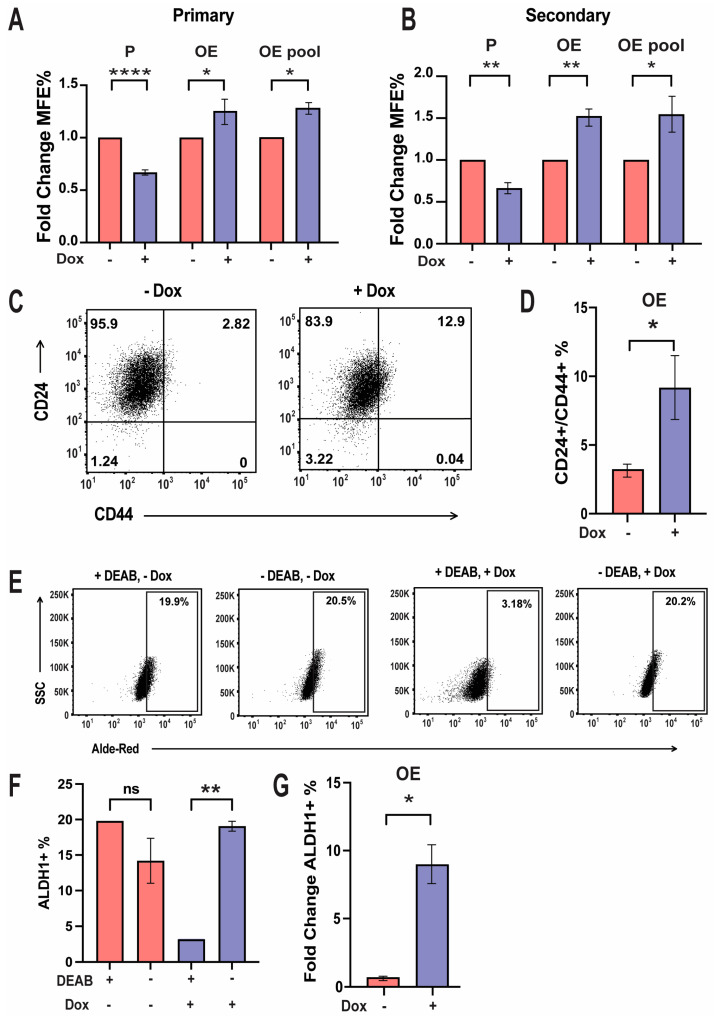
GRHL2 overexpression enriches stem cell-like characteristics. (**A**) Quantification of primary of P, OE, and OE pool cells. GFP immunofluorescence microscopy confirmed GRHL2 induction after initial Dox treatment. Error bars represent the mean fold change in mammosphere formation efficiency (MFE%) relative to vehicle. n = 4. *, *p* < 0.05; ****, *p* < 0.0001. (**B**) Quantification of secondary P, OE, and OE pool cells. GFP immunofluorescence microscopy confirmed GRHL2 induction after initial Dox treatment. Error bars represent the mean fold change in mammosphere formation efficiency (MFE%) relative to vehicle. n = 4. *, *p* < 0.05; **, *p* < 0.01. (**C**) Representative flow cytometry profiles of CD24 and CD44 expression in OE cells. Numbers refer to % of cells in the population. n = 3. (**D**) Quantification of flow cytometry analyses on % of cells co-expressing CD24 and CD44 in OE cells. Error bar represents the mean CD24+/CD44+ % ± SEM. n = 3. *, *p* < 0.05. (**E**) Representative flow cytometry profiles of ALDH1 activity in OE cells using the Aldefluor assay. SSC refers to the side scatter optical detector. Gating represents the % of ALDH1-positive cells in the OE population. Diethylaminobenzaldehyde (DEAB) was used as a control for the background signal. (**F**) Quantification of the % of OE cells expressing ALDH1. Error bar represents the mean ALDH1+ % ± SEM. n = 3 for DEAB-negative cells. **, *p* < 0.01. ns = not significant. (**G**) Quantification of fold change ALDH1+ % in OE cells as compared to the DEAB control. Error bars represent the mean ALDH % ± SEM relative to DEAB control. n = 3. *, *p* < 0.05.

Breast cancer progression can involve long periods of dormancy, as well as selection for stem cell characteristics [59,60]. Primary mammosphere formation assays were next assessed to determine if high levels of GRHL2 promoted self-renewal of breast cancer cells [61,62]. While P cells were incapable of forming sufficient mammospheres after 14 days of incubation, OE cells and an OE pool overexpressing GRHL2 significantly increased the size and numbers of mammospheres as compared to no Dox treatment (Figure 6A). Proper GRHL2 induction with initial Dox treatment was confirmed by immunofluorescence microscopy of primary mammospheres at time of mammosphere formation efficiency (MFE%) analysis (see Supplementary Figure S2A). Secondary mammosphere formation assays were initiated by dissociating pelleted primary mammospheres and re-seeding single cells in mammosphere medium to further observe the capacity for self-renewal. Likewise, secondary mammospheres recapitulated the primary mammospheres, with GRHL2 overexpression giving rise to an increased number of secondary mammospheres as compared to no Dox treatment (Figure 6B). P cells could not form secondary mammospheres to the size of the OE and OE pool cells (see Supplementary Figure S2A).

To further observe stem cell enrichment induced by GRHL2 overexpression, we utilized FACS to visualize the representation of cell surface adhesion protein CD44. CD44 has been widely studied as a marker for stem cells with links to promoting metastasis in breast cancer [63,64]. OE cells treated with Dox for 72 h showed increased co-expression of CD24 and CD44 markers as compared to cells with no Dox (Figure 6C). A larger percentage of the population occupied the CD24+/CD44+ space, denoting simultaneous expression of stem cell-related, and more broadly metastasis-related, markers [65]. MDA-MB-231 cells were used for gating purposes to ensure consistent CD44+ parameters (see Supplementary Figure S2B). FACS profiles of P cells revealed no change in CD24 and CD44 co-expression with and without Dox treatment (see Supplementary Figure S2C). As an orthogonal approach, MCF7 cells transiently transfected with a GRHL2-GFP plasmid were used to further corroborate effects of elevated GRHL2 on stem cell enrichment. After 48 h, FACS profiles revealed an increased CD24+/CD44+ population as compared to a vector control (see Supplementary Figure S2D). Quantification of FACS profiles revealed a significant increase in the percentage of cells co-expressing CD24+/CD44+ markers in the population as compared to no Dox treatment (Figure 6D). These double-positive cells in the population characterize a cancer stem cell phenotype induced by elevated GRHL2.

ALDH1 activity is another marker used to characterize stem cell behavior in various tissues, with the specific isoform ALDH1α elevated in stem cells and progenitor cancer stem cells [66]. OE cells treated with Dox exhibited an increased percentage of cells expressing ALDH1 in the population when compared to no Dox (Figure 6E). Diethylaminobenzaldehyde (DEAB) was used as a control for the background signal to inhibit ALDH1 activity. MDA-MB-231 cells were used for gating purposes to ensure ALDH1+ expression (see Supplementary Figure S3A). Quantification of the percent of cells expressing ALDH1+ in OE cells attests to the false ALDH1+ expression in OE cells not treated with Dox and the true ALDH1+ expression in OE cells treated with Dox as DEAB could not inhibit ALDH1 expression in vehicle-treated cells (Figure 6F). Therefore, we quantified the fold change in the ALDH1+ percentage in OE cells as compared to the DEAB control to demonstrate an increased ALDH1+ expression in line with the restrictions of the Aldefluor assay. This affirms the enrichment of a stem cell phenotype with elevated GRHL2 (Figure 6G). FACS profiles of MCF7 cells transiently transfected with a GRHL2-GFP plasmid similarly had an increased ALDH1+ population when compared to a vector control (see Supplementary Figure S3B). Hence, ER-positive cells with elevated GRHL2 through either an inducible or transient model increase ALDH1 expression.

Taken together, these results show that GRHL2 overexpression enriches the stem cell characteristic of self-renewal and increases cell surface marker CD44 as well as ALDH1. Through the increase in CD44 and ALDH1 protein expression, GRHL2 stimulates the enrichment of cancer stem cells in the population [67].

### 3.7. GRHL2 Overexpression Leads to a Complex Epithelial–Mesenchymal Hybrid Phenotype

**Figure 7:**
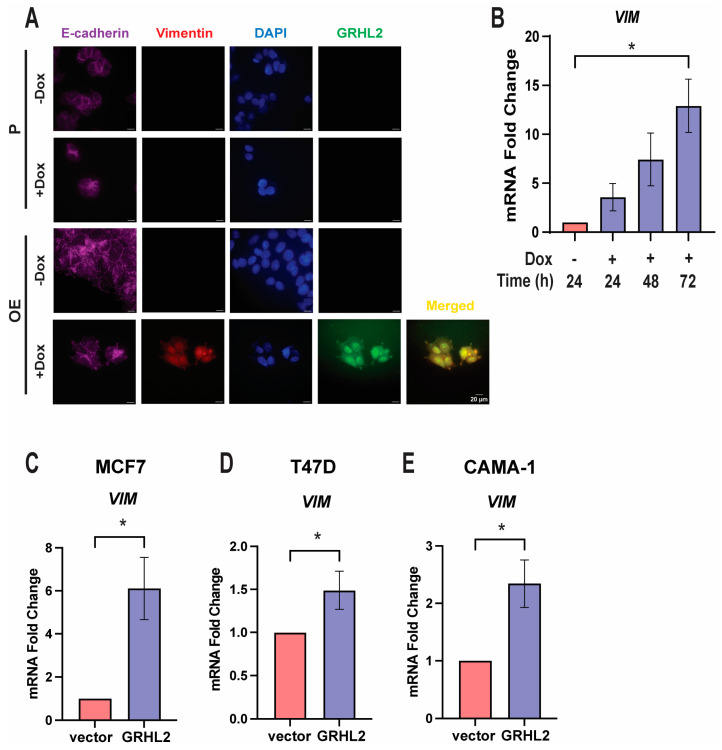
GRHL2 overexpression leads to a complex epithelial–mesenchymal hybrid phenotype. (**A**) Fluorescence microscopy immunocytochemistry of E-cadherin and vimentin in P and OE cells treated with 1 μg/mL Dox for 72 h. Fluorescence imaging attained by 600× oil microscopy with 0.33 μm/pixels, scale bar of 20 μm. (**B**) RT-qPCR analysis of *VIM* in OE cells treated with 1 μg/mL Dox and harvested at the indicated times. Error bars represent the mean mRNA fold change ± SEM relative to the vehicle. n = 3. *, *p* < 0.05. (**C**) RT-qPCR analyses of *VIM* mRNA in MCF7 cells transiently transfected with *GRHL2-GFP* DNA. Error bars represent the mean mRNA fold change ± SEM relative to the vector control. n = 3. *, *p* < 0.05. (**D**) RT-qPCR analyses of *VIM* mRNA in T47D cells transiently transfected with *GRHL2-GFP* DNA. Error bars represent the mean mRNA fold change ± SEM relative to the vector control. n = 3. *, *p* < 0.05. (**E**) RT-qPCR analyses of *VIM* mRNA in CAMA-1 cells transiently transfected with *GRHL2-GFP* DNA. Error bars represent the mean mRNA fold change ± SEM relative to the vector control. n = 3. *, *p* < 0.05.

Given that high levels of GRHL2 were inducing multiple phenotypes associated with breast cancer progression, yet also enriching epithelial markers, we asked if a high GRHL2 expression may induce a plastic state observed during EMT. Immunocytochemistry (ICC) staining for E-cadherin, vimentin, and GRHL2 was completed to confirm protein expression of epithelial and mesenchymal identity markers. OE cells treated with 1 μg/mL Dox for 72 h co-expressed E-cadherin and vimentin, emphasizing the induction of a hybrid state in which cells express not only epithelial markers, but also markers associated with mesenchymal cells (Figure 7A). Thus, elevated GRHL2 induces a complex hybrid phenotype not present in P cells or untreated OE cells. Antibody specificity for E-cadherin and vimentin was confirmed using MCF7 and MDA-MB-231 cells, respectively (see Supplementary Figure S4A,B). A similar EMT hybrid state can be confirmed by ICC using overexpressing GRHL2 cells in an OE pool, and MCF7 and T47D cells transiently transfected with 1 μg GRHL2-GFP plasmid for 48 h (see Supplementary Figure S4C–E).

The EMT hybrid state was corroborated by analysis of *VIM* mRNA expression in OE cells treated with Dox for varying lengths of time. Vimentin expression increased in a dose-dependent manner equivalent to the increase in GRHL2 expression as compared to no Dox (Figure 7B). Isolated RNA from OE pools treated with 1 μg/mL Dox for 72 h similarly showed an increase in *VIM* expression (see Supplementary Figure S4F). MCF7, T47D, and CAMA-1 cells transiently transfected with 1 μg GRHL2-GFP plasmid similarly showed significantly increased *VIM* expression as compared to a vector control (Figure 7C–E). ChIP qPCR also confirmed an increase in H3K27 acetylation for active transcription at the vimentin promoter under similar conditions (see Supplementary Figure S4G). Together, these results suggest that elevated GRHL2 levels allow cells to enter into a plastic EMT state with the ability to maintain both epithelial and mesenchymal traits. The presence of an EMT hybrid state induced by high GRHL2 expression is consistent with dormancy and the enrichment of stem cell-like characteristics in breast cancer cells [68,69].

### 3.8. GRHL2 Overexpression Alters GRHL2 Genome Binding in a Dynamic Manner

**Figure 8:**
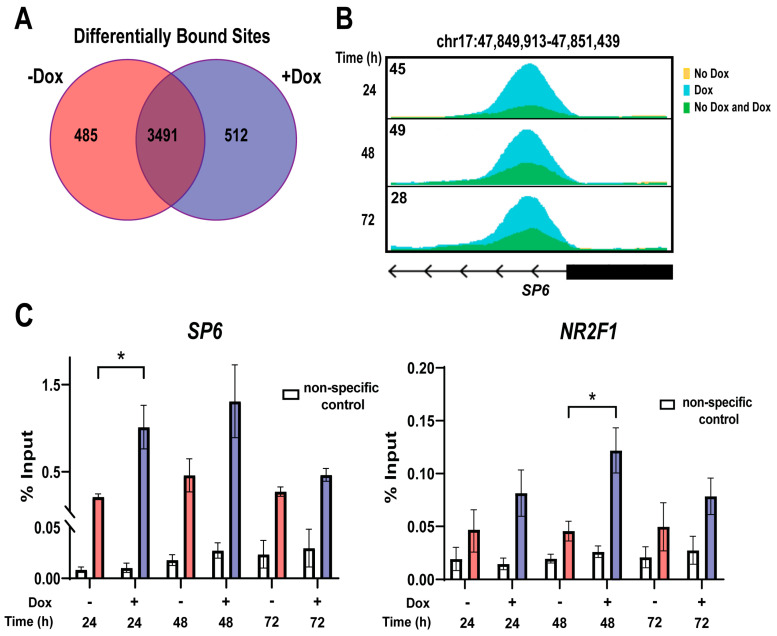
GRHL2 overexpression alters GRHL2 genome binding in a dynamic manner. (**A**) Venn diagram displaying differentially bound sites between +Dox and −Dox datasets in GRHL2-overexpressing OE cells. −Dox and +Dox datasets represent the overlap of 24, 48, and 72 h datasets under −Dox and +Dox conditions, respectively. Three separate binding groups were established: −Dox only (485 sites), Dox independent (3481 sites), and +Dox only (512 sites). (**B**) Representative genome track of a GRHL2 binding site. Internal numbers represent ChIP signal intensity. (**C**) RT-qPCR analysis of *SP6* and *NR2F1*, a representative +Dox binding group and dormancy gene, respectively, under −Dox (red) or +Dox (blue) treatment at the indicated times. Error bars represent the mean ± SEM, n = 3. *, *p* < 0.05.

The emergence of altered gene expression reflective of a hybrid EMT state indicates changes in GRHL2 behavior at the genomic level upon overexpression. ChIP-seq for GRHL2 was performed in OE cells with and without Dox for 24, 48, or 72 h. High confidence untreated (−Dox) and GRHL2-overexpressing (+Dox) peak sets were generated by taking only the top 5000 peaks from the total GRHL2 binding sites found at each time point, then determining which of those peaks were present at all time points. Differential peak analysis, performed by merging the high confidence −Dox and +Dox peak sets, revealed 485 binding sites specific to the −Dox condition, 512 binding sites specific to the +Dox condition, and 3491 shared binding sites (Figure 8A, see Supplemental Table S3). A representative binding site near the *SP6* promoter shows that while GRHL2 is bound at this site prior to treatment, Dox treatment induces a greater signal intensity that changes over time (Figure 8B). This finding was confirmed by qPCR, which showed a significant increase in binding intensity with Dox treatment (Figure 8C). Similarly, significantly higher binding intensity was observed at dormancy gene *NR2F1* (Figure 8C). The binding intensity at both genes returns to roughly baseline levels at 72 h. Taken together, these analyses show that GRHL2 binds with more intensity when overexpressed, and that this binding also fluctuates with time.

### 3.9. GRHL2 Overexpression Changes Motifs Found near GRHL2 Binding Sites

**Figure 9:**
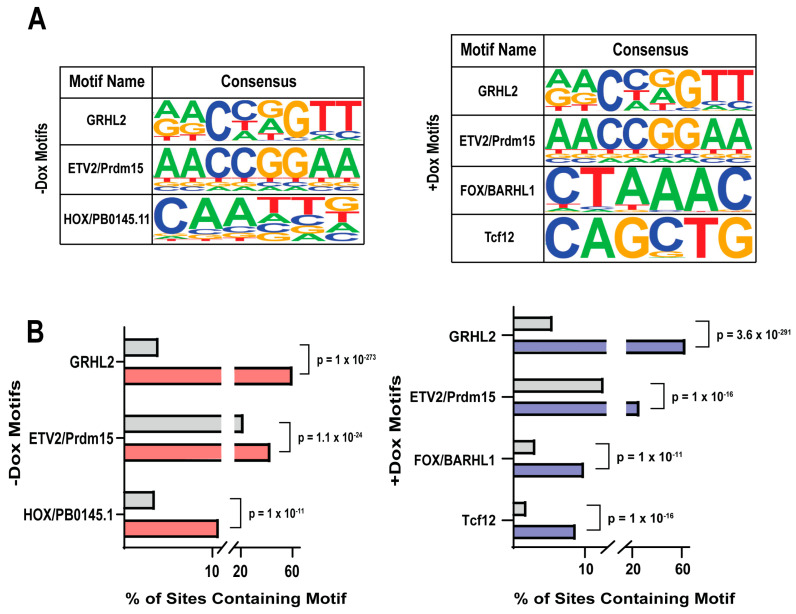
GRHL2 overexpression changes motifs found near GRHL2 binding sites. (**A**) Consensus sequence logos representing the top motifs in the −Dox and +Dox peak sets, acquired from HOMER de novo motif analysis. (**B**) Motif analyses using top motifs from −Dox (red) and +Dox (blue) peak sets relative to background (gray). Data are shown as % of binding sites that contain the specific motif. *p*-values are derived from chi-square test and HOMER de novo motif analysis.

De novo motif analysis was conducted on GRHL2 binding sites in the −Dox and +Dox peak sets (Figure 9A). As expected, the most commonly found motif in both analyses was GRHL2 (Figure 9B). Motifs found near the endogenous GRHL2 binding sites in untreated cells showed high congruence to those that bind transcription factors associated with differentiation and development, such as ETV2 and HOX. ETV2 plays an important role in endothelial cell differentiation, while HOX genes are well known for their significance in fetal development [70,71]. The presence of these motifs near GRHL2 binding sites in untreated cells is in line with the well-established role of endogenous GRHL2 in facilitating cellular development [9,12,13]. When GRHL2 levels are high, there is a shift in motifs. While GRHL2 motifs remain the most common, there is the appearance of motifs similar to those of FOX and Tcf12 transcription factors; transcription factors linked to initiation of EMT, endocrine therapy resistance, and invasion in breast cancer [72,73,74]. This shift in binding site usage, combined with the gene expression and phenotypic changes associated with GRHL2 overexpression, indicates that high levels of GRHL2 alter its transcription factor activity to promote gene expression changes resulting in hybrid EMT, stem cell, and dormancy-promoting phenotypes.

## 4. Discussion

GRHL2 plays a dynamic role with tumor-suppressive and oncogenic functions in a context-dependent manner [21,46,47,75,76]. While the loss of GRHL2 is implicated in increased migratory behavior commonly associated with metastasis in ER-positive breast cancer cells, high levels of GRHL2 expression often occur in ER-positive breast cancer cells and are associated with poor overall and distant metastasis-free survival in breast cancer patients [7,16,26]. Here, we showed that ER-positive breast cancer cells overexpressing GRHL2 significantly increased epithelial markers and behavior as compared to those with only endogenous levels of GRHL2, indicating that GRHL2 retains its potent role in epithelial identity when expressed at high levels. However, RNA sequencing revealed that elevated levels of GRHL2 are also linked to pathways commonly associated with EMT and stem cell characteristics. At high levels, GRHL2 begins to regulate transcription of genes associated with dormancy, stemness, and EMT plasticity, highlighting a pleiotropic role in tumor progression. Altered DNA binding suggested that biological changes associated with GRHL2 overexpression may stem from changes in cooperating transcription factors at the genomic level. These observations led us to propose a model in which high levels of GRHL2 are able to push cells toward a hybrid EMT state exhibiting both epithelial and mesenchymal cell traits. This plasticity changes cellular identity and stimulates dormancy, stemness, and altered DNA binding.

We show that elevated GRHL2 in an ER-positive breast cancer cell background resulted in the simultaneous expression of both epithelial and mesenchymal markers in the same cell. This is in line with GRHL2 studies in pancreatic and ovarian models [46,77]. This is referred to as a hybrid EMT state, which is known to be directly related to disease aggressiveness [78]. Though EMT can be articulated as a distinctive transition between its binary states, a cell’s capacity to become plastic and express a hybrid EMT state with both epithelial and mesenchymal markers re-defined the literature’s understanding of metastasis [79,80,81]. Since then, this dynamic process has been shown to promote tumorigenic properties with increased therapy resistance, reduced survival, and cellular reprogramming in pancreatic, human mammary epithelial, and ovarian cancer cells [82,83,84]. Early work by Jolly et al. proposed a theoretical model in which the coupled relationship of GRHL2 and other factors can collectively stabilize a transient EMT hybrid state [79]. Our work expands on this original hypothesis in that elevated levels of GRHL2 alone can induce plasticity. Previous work by Bai et al. suggested that expression of GRHL2 induced a hybrid EMT phenotype in breast cancer cells. While this study similarly utilizes an overexpressing GRHL2 ER-positive MCF7 cell line to observe EMT marker expressions, vimentin expression was present in control cells and further decreased with GRHL2 overexpression, as assessed by Western blotting [26]. Our work showed GRHL2 overexpression only induced vimentin expression following a long-term 72 h Dox treatment, as shown by ICC and RT-qPCR. The former study also used an ER-negative model which lacks endogenous GRHL2 expression and which has inherent mesenchymal phenotypes, whereas we sought to elevate the levels of GRHL2 protein in an ER-positive cell with a true epithelial background [26]. In contrast, our work further emphasizes elevated GRHL2 induced epithelial strengthening with a decrease in migration and upregulation of known epithelial identity markers *CDH1* and *CLDN4*. The combinatorial expression of epithelial and mesenchymal markers in elevated GRHL2 ER-positive cells demonstrates the capacity for ER-positive cells to gain the plasticity needed to overcome the non-native environments that breast cancer cells encounter during the metastatic cascade, while also strengthening the epithelial phenotype. Recent evidence indicates that secondary, metastatic tumors are more similar to original, epithelial cells achieving a hybrid EMT state rather than a complete EMT to mesenchymal to epithelial (MET) transformation [85,86,87]. Fischer et al. and their usage of lineage tracing also showed that complete EMT is not required for lung metastases, and that tumor cells persist in their epithelial phenotypes while disseminating and forming metastases in lung cancer [87]. Hence, elevated levels of GRHL2 could support ER-positive breast cancer progression by supporting plastic, resilient tumor-initiating cells [88].

Interestingly, high GRHL2 expression increases stem cell markers and self-renewal characteristics in a luminal breast cancer cell, as well as markers of dormancy. Increasing evidence points to the interplay of cellular heterogeneity and the aptness for cancer cells to respond to their environment by acquiring properties associated with stem cell and dormancy behaviors [67,81,89,90,91,92]. Our work illustrated an enrichment in self-renewal capacity with an increase in mammospheres formed with elevated GRHL2 expression in ER-positive breast cancer cells. These same cells exhibited a shift from a CD24+/CD44− population with no ALDH1+ expression to a co-expressing CD24+/CD44+ phenotype and increasing expression of ALDH1, highlighting the presence of stem cell-related markers with high GRHL2 expression. Each of these phenotypes jointly articulate stem cell-like enrichment and plasticity imparted by elevated GRHL2 levels in an ER-positive breast cancer cell to perpetuate tumor progression [67,93,94]. Our examination of elevated GRHL2 on stem cell activity supports the mathematical model proposed by Mooney et al., which speculated that GRHL2 could indirectly regulate CD24 and CD44 to support a hybrid, co-expressing CD24 and CD44 state [11]. We also observed an increase in dormancy markers *NR2F1* and *CDKN1B* and a slowing of proliferation and tumor growth in vivo with GRHL2 overexpression. This is consistent with the persistent, long-term dormancy seen in head and neck squamous cell carcinomas and breast cancer cell lines, where dormant disseminated tumor cells are characterized by upregulation of these key dormancy genes and decreased proliferation markers [95,96]. In excised murine tumors, IHC revealed an increase in dormancy gene *CDKN1B*-encoded protein p27 and a decrease in proliferation marker Ki67, recapitulating the inverse expression observed in quiescent cancer cells [97]. Of particular note, the composition of the cell cycle was not influenced by increased expression of genes associated with cellular dormancy. This emphasizes how elevated GRHL2 can sustain a non-proliferating, non-apoptotic cell in dormancy. The link between GRHL2 overexpression and dormancy is especially relevant considering ER-positive breast cancer has a lower long-term survival rate than hormone receptor-negative breast cancer [56,98]. Long-term tumor recurrence can occur more than a decade after primary tumor treatment, indicating an extended period of dormancy [99,100]. Dormancy and cancer stem cell behaviors are known to be associated with tumor progression [89,96,101,102]. Weidenfeld and Barkan suggest that a dormant tumor cell can distinctively possess both self-renewal capacity and quiescence to evolve based on cues from the microenvironment [103]. Elevated GRHL2 can play a disproportionate role in breast cancer progression with the coexistence of stem cell and dormancy behaviors supporting cellular plasticity for tumor initiation and resistance for ER-positive breast cancer cells.

In order to ascertain whether the phenotypic changes were a result of altered GRHL2 binding to DNA, we conducted ChIP-seq. Previous groups have done ChIP-seq in breast cancer cells [7,20,22,24,25,104]. In contrast, here we examine how increases in breast cancer cell GRHL2 protein concentration altered DNA binding. Concentration-dependent, “condition-expanded” activation of transcription has been reported for ER in breast cancer [105], as well as for other classes of transcriptional regulators in Drosophila [106,107] and yeast [108]. High confidence peak sets were created by compiling all GRHL2 binding peaks at three time points, then selecting only the top 5000 peaks that were common to all time points. The resulting high confidence peak sets were used for differential peak analysis and showed that elevated GRHL2 resulted in increased binding at 512 sites and decreased binding at 485 sites. Representative sites that were increased with GRHL2 overexpression, *NR2F1* and *SP6*, exhibited differences in GRHL2 binding over time. The presence of increased GRHL2 binding near the dormancy gene *NR2F1* complements the increased *NR2F1* expression observed in our gene expression analysis, as well as the lack of proliferation observed in the murine models. The *SP6* gene was recently linked to an increased risk of prostate cancer, as well as to more aggressive disease [109]. The strongest changes in binding were found after 24 and 48 h following induction of GRHL2 overexpression, with a return to basal levels by 72 h. The dynamic change in GRHL2 binding cannot be due to changes in GRHL2 protein levels, since GRHL2 levels remain high at 72 h as seen by both Western and ICC analyses. However, it is possible that at late time points, the GRHL2 epitope could be masked by other transcription factors as part of transcriptional complexes. Separate from its role as a transcription factor, GRHL2 is also known to possess pioneer factor functions in both *Drosophila* and mammals [110,111,112]. Studies by Gibson et al. with Grh, the *Drosophila* analog of mammalian GRHL, determined that Grh chromatin binding was concentration-dependent, with binding increasing with protein concentration. Increased levels of Grh were found to be sufficient to open chromatin at certain Grh binding sites that were inaccessible at physiological levels [113]. Further, their studies suggest that protein concentration and affinity for the binding site can affect pioneer function. Therefore, the changes in DNA binding with elevated GRHL2 could result from either changes in its transcription or pioneer factor function.

We conducted a de novo motif analysis on the motifs surrounding GRHL2 binding sites from cells with endogenous or elevated levels of GRHL2. Peaks identified in the presence of endogenous levels of GRHL2 featured nearby ETV2 and HOX motifs associated with growth and development [70,71]. Alternatively, binding sites in cells expressing high levels of GRHL2 showed nearby FOX and Tcf12 motifs, which are associated with tumor progression in the context of breast cancer [72,73]. Members of the FOX transcription factor family, specifically FOXA1, have been implicated in drug resistance in ER-positive breast cancer cells. A study by Cocce et al. found a significant increase in FOXA1 binding sites in tamoxifen-resistant cells. The tamoxifen-resistant model used in that study had elevated levels of GRHL2 when compared to tamoxifen-sensitive cells, emphasizing the relationship between increased GRHL2, FOXA1, and hormone therapy resistance [24]. Interestingly, while we similarly observed an increase in FOXA1 motifs at high levels of GRHL2, our studies indicate that GRHL2 binds near motifs associated with development in hormone-sensitive breast cancer cells. This indicates that elevated GRHL2 may be a key driver of new binding sites that change from development to therapy-resistant programs. This agrees with studies by Kumegawa et al. in primary breast cancer patient data showing that GRHL2 binding sites in luminal breast cancer tumors with an enriched GRHL2 motif were associated with genes implicated in tumor progression [114].

There are some limitations with this study. While the use of cell line models allows for a better understanding of the causal link between GRHL2 and poor clinical prognosis, confirmation using patient-derived models would provide further validation. Investigation of the impact of the 3D tumor microenvironment using tumor spheroid or organoid systems and patient-derived xenografts in humanized mouse models would complement the mammosphere and tumor xenograft models used herein. Follow-up studies employing genomic/proteomic profiling and CRISPR screens could help dissect GRHL2 downstream effectors and biology. Lastly, the current study focused on the effects of high levels of GRHL2 in the context of ER-positive breast cancer and did not include other molecular subtypes or analysis of the immune landscape which may be impacted by the EMT signature [115]. The potential influence of GRHL2 on the tumor immune response and regulation of programmed death-ligand 1 (PD-L1) warrants investigation [116,117,118].

Future studies can also focus on the clinical implications of these results such as investigating GRHL2 as an actionable biomarker in ER-positive breast cancer. Integrating GRHL2 status into pathologic evaluation and clinical decision making may help individualize breast cancer treatment based on the potential to activate dissemination programs through this plasticity factor. The development of GRHL2-directed therapeutic agents would be a new approach to test in combination with endocrine therapy.

## 5. Conclusions

In summary, this work supports the notion that elevated levels of GRHL2 in ER-positive breast cancer cells sustain an epithelial cell identity while facilitating a hybrid EMT state and inducing stem cell and dormancy markers. These properties confer a subset of cells in the population with traits associated with metastatic and tumor-initiating cells. As an endogenous transcription factor, GRHL2 binds to consensus GRHL2 binding sites throughout the genome. However, at high levels, GRHL2 preferentially binds sites associated with motifs of transcription factors associated with breast cancer progression. Together, these findings indicate that elevated levels of GRHL2 in ER-positive breast cancer cells can alter its DNA binding activity to promote the expression of genes implicated in a plastic, hybrid state that is associated with increased metastatic potential.

## Data Availability

The original data presented in this study are openly available in the Gene Expression Omnibus (GEO) database at Home-GEO-NCBI under accession number GSE272059 and GSE272061.

## Supplementary Materials

The following supporting information can be downloaded at: https://www.mdpi.com/article/10.3390/cancers16162906/s1, Supplementary Figure S1: High GRHL2 expression increases epithelial cell phenotypes, Supplemental Table S1: GRHL2 overexpression alters its endogenous transcriptional activity and gene expression, Supplementary Figure S2: Transient GRHL2 overexpression yields stem cell enrichment, Supplementary Figure S3: Transient GRHL2 overexpression increases ALDH1 expression, Supplementary Figure S4: GRHL2 overexpression induces an EMT hybrid state at the protein and genomic levels, Supplemental Table S2: Flow cytometry cell cycle analysis of percentage of cells in G1, G2, and S phase, Supplemental Table S3: GRHL2 overexpression alters GRHL2 genome binding in a dynamic manner. Uncropped Western blots are included as well.
