# Supplementary material for: Elevated GRHL2 Imparts Plasticity in ER-Positive Breast Cancer Cells
Source: Cancers (Basel). 2024 Aug 21;16(16):2906. doi: 10.3390/cancers16162906 (PMC11353109; doi:10.3390/cancers16162906)
Supplement: Supplementary file 1 [file cancers-16-02906-s001.zip › cancers-3138613-supplementary.pdf]

Supplemental Table 2: Flow cytometry cell cycle analysis of percentage of cells in G1, G2, and S phase.

			G1 (%)	G2 (%)	S (%)
**24 h**	**P**	**no Dox**	46.4 ± 1.7	16.1 ± 2.2	37.5 ± 3.1
		**Dox**	48.1 ± 0.8	20.3 ± 0.7	31.6 ± 0.3
	**OE**	**no Dox**	53.5 ± 1.4	19.1 ± 3.9	27.4 ± 0.4
		**Dox**	55.9 ± 1.3	17.8 ± 1.9	26.3 ± 0.3
**48 h**	**P**	**no Dox**	63.3 ± 0.3	19.7 ± 1.6	17.0 ± 4.2
		**Dox**	54.7 ± 1.0	11.8 ± 0.5	33.5 ± 2.5
	**OE**	**no Dox**	52.4 ± 3.7	23.6 ± 2.4	24.1 ± 2.5
		**Dox**	59.0 ± 0.2	13.5 ± 1.2	27.5 ± 0.5
**72 h**	**P**	**no Dox**	67.4 ± 3.3	17.2 ± 2.0	15.4 ± 3.7
		**Dox**	70.1 ± 1.0	10.0 ± 0.9	19.9 ± 2.8
	**OE**	**no Dox**	57.5 ± 2.0	16.6 ± 2.4	25.9 ± 2.4
		**Dox**	54.7 ± 0.7	17.3 ± 1.0	27.9 ± 1.3

Mean ± SEM; P=parental, OE=Overexpress, h=hour.

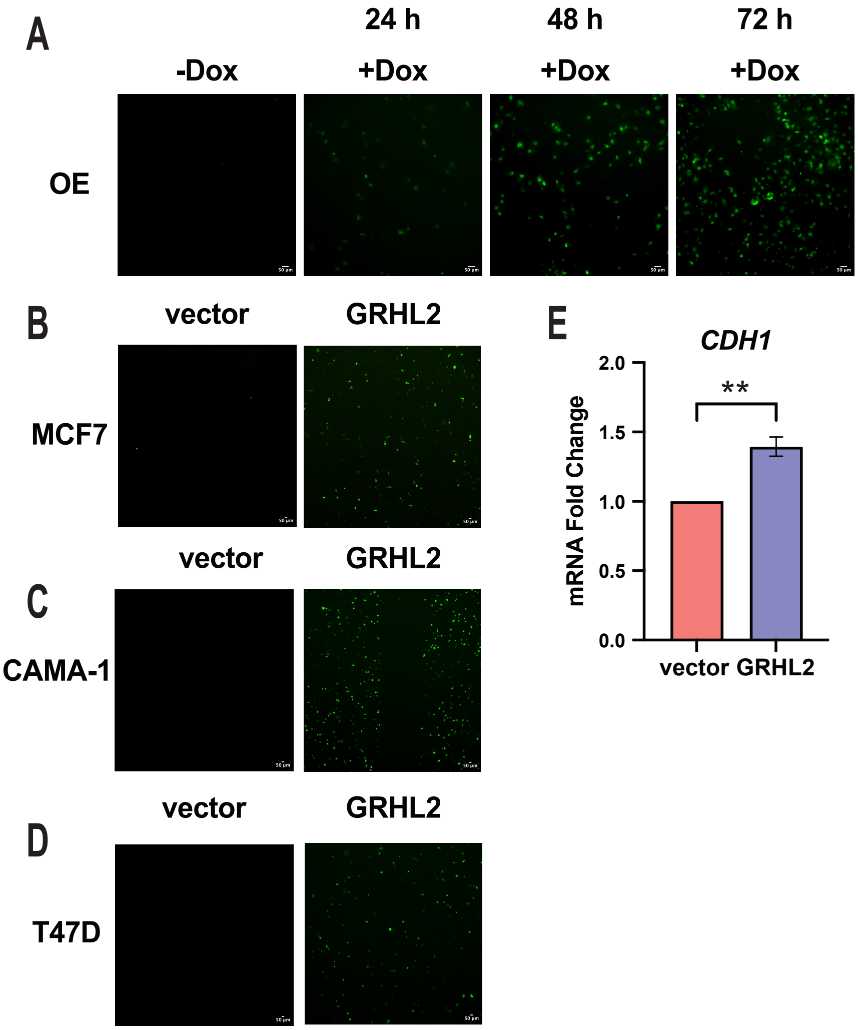

Supplementary Figure S1. (Related to Figure 2): High GRHL2 expression increases epithelial cell phenotypes. **(A)** Representative GFP fluorescence microscopy of OE cells. GFP signal is indicative of GRHL2-GFP induction. Fluorescence imaging attained by 100x microscopy with 0.65 µm/pixels, scale bar of 50 µm. Representative GFP fluorescence microscopy of MCF7 **(B)**, CAMA-1 **(C)**, and T47D **(D)** cells transiently transfected with GRHL2 or vector control DNA. Fluorescence imaging attained by 40x microscopy with 1.63 µm/pixels, scale bar of 50 µm. Images of CAMA-1 cells were supplemented from migration assay acquisition. **(E)** RT-qPCR analysis of CDH1 in T47D cells transiently transfected with GRHL2 DNA. Error bar represents the mean mRNA fold change ± SEM relative to the vector control. n = 4. **, $p < 0.01$ as determined by a paired *t*-test.

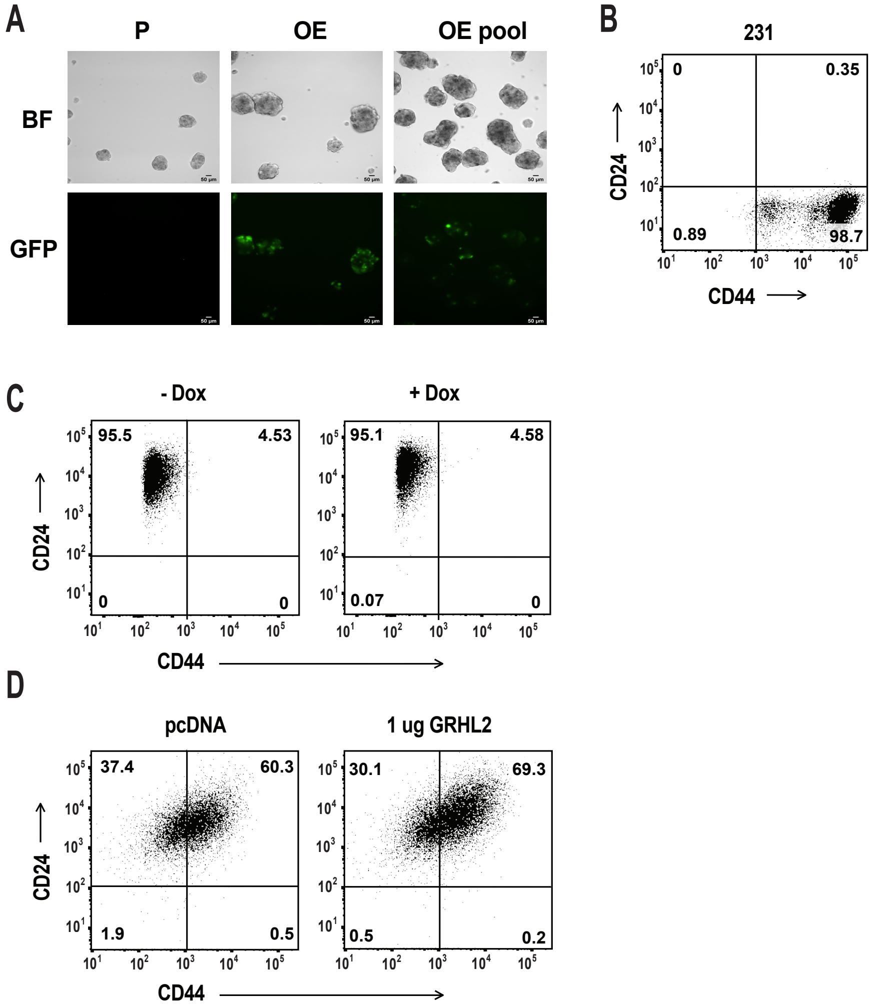

Supplementary Figure S2. (Related to Figure 6): Transient GRHL2 overexpression yields stem-cell enrichment. **(A)** Representative GFP fluorescence microscopy of P, OE and OE pool mammospheres at time of MFE% analyses. Fluorescence imaging attained by 100x microscopy with 0.65 µm/pixels, scale bar of 50 µm. **(B)** Flow cytometry profiles of CD24 and CD44 expression in MDA-MB-231 cells used to set gating of CD44 positive parameters. **(C)** Representative flow cytometry profiles of CD24 and CD44 expression in P cells. Numbers refer to the % of cells in the population. **(D)** Representative flow cytometry profiles of CD24 and CD44 expression in MCF7 cells transiently transfected with GRHL2 or vector control DNA and subjected to FACS sorting. Numbers refer to the % of cells in the population.

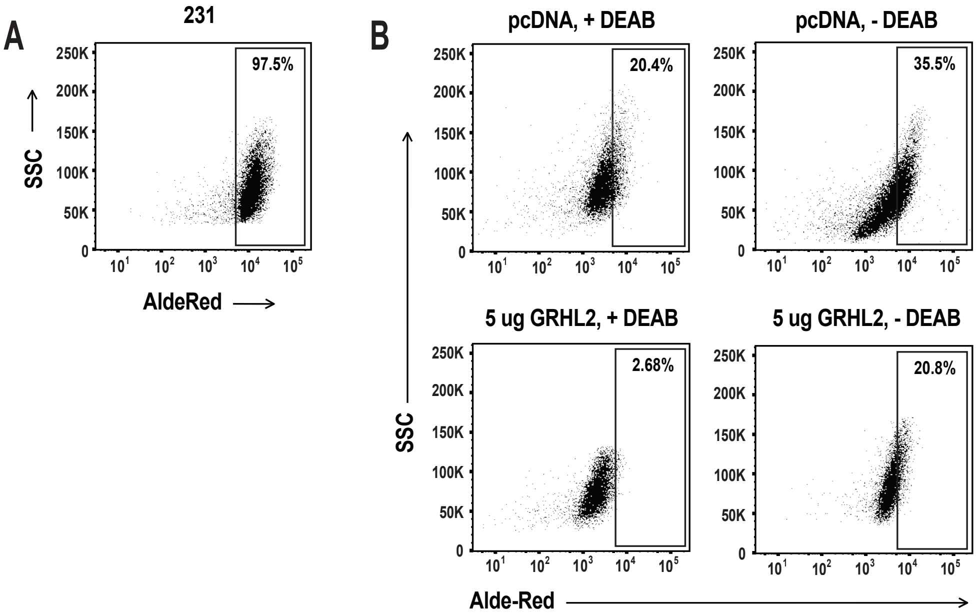

Supplementary Figure S3. (Related to Figure 6): Transient GRHL2 overexpression increases ALDH1 expression. **(A)** Flow cytometry profile of ALDH1 expression in MDA-MB-231 cells used to set gating of ALDH1 positive parameters. **(B)** Representative flow cytometry profiles of ALDH1 activity in MCF7 cells transiently transfected with GRHL2 or vector control DNA (pcDNA) prior to FACS sorting using the Alde-Fluor assay. Gating represents the % of ALDH1 positive cells in the population. DEAB was used as an internal control.

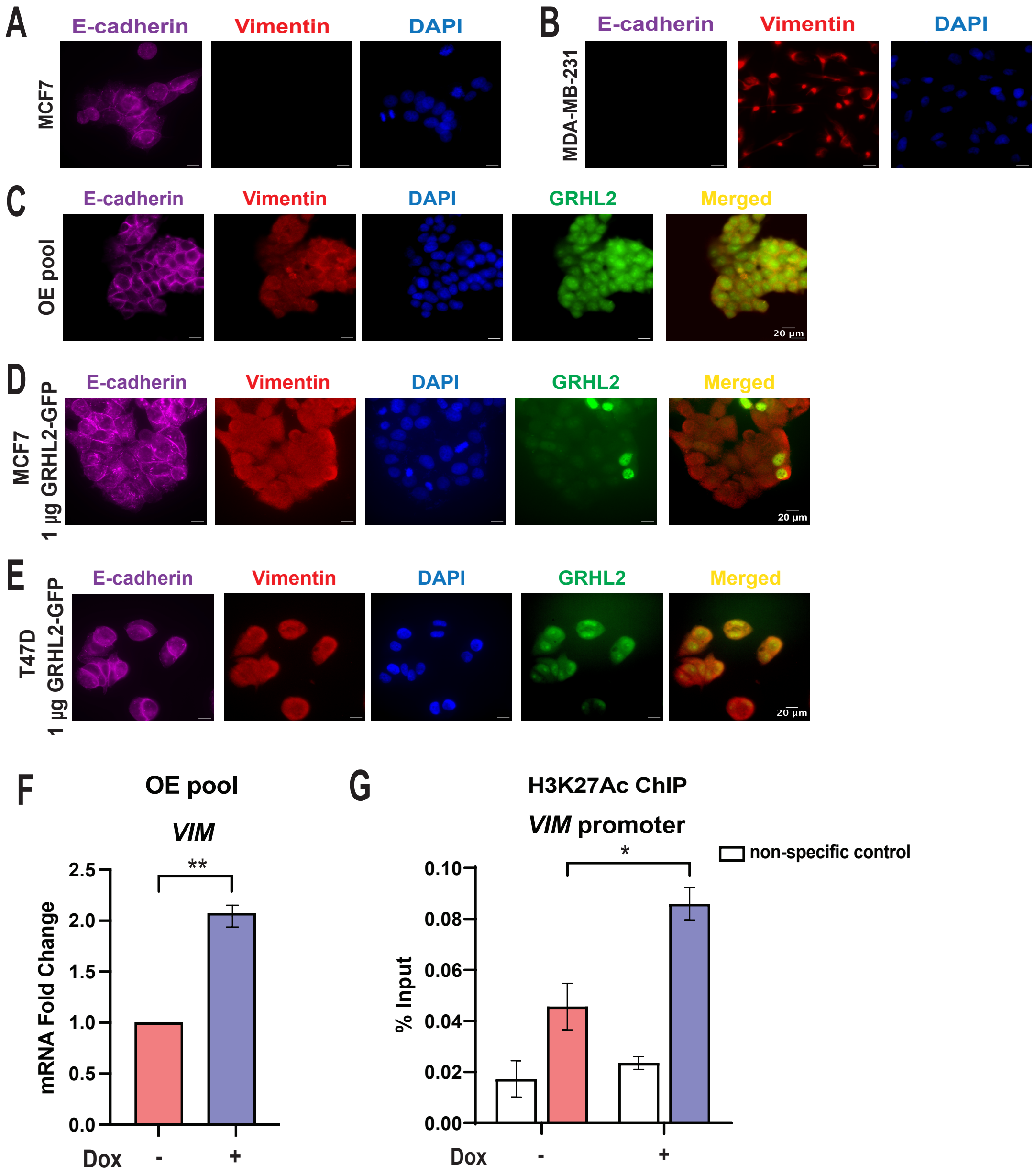

Supplementary Figure S4. (Related to Figure 7): GRHL2 overexpression induces an EMT hybrid state at the protein and genomic levels. Representative immunocytochemistry fluorescence microscopy of MCF7 **(A)** and MDA-MB-231 **(B)** cells shown to confirm E-cadherin and vimentin antibody specificity. Similar immunocytochemistry staining for E-cadherin and vimentin were completed in OE pool **(C)** and MCF7 **(D)** and T47D **(E)** transiently transfected with GRHL2 DNA. Fluorescence imaging attained by 600x oil microscopy with 0.33 µm/pixels, scale bar of 20 µm. **(F)** RT-qPCR analysis of VIM in the OE pool. Error bar represents the mean mRNA fold change ± SEM relative to the vehicle. n = 3. **, p < 0.01 as determined by a paired *t*-test. **(G)** ChIP RT-qPCR analysis of H3K27 acetylation at the VIM promoter in OE cells. Data are reported as a percentage of input. A non-specific genomic site was utilized as a control (white). Error bars represent the mean ± SEM. n = 3. *, p < 0.05 as determined by a paired *t*-test.
